# Effectiveness of cognitive-behavioral therapy on sexual function and sexual self-efficacy in pregnant women: An RCT

**DOI:** 10.18502/ijrm.v13i8.7504

**Published:** 2020-08-19

**Authors:** Mina Nezamnia, Mina Iravani, Mehdi Sayah Bargard, Mahmood Latify

**Affiliations:** ^1^Student Research Committee, Department of Midwifery, Nursing and Midwifery School, Ahvaz Jundishapur University of Medical Sciences, Ahvaz, Iran.; ^2^Reproductive Health Promotion Research Center, Department of Midwifery, Nursing and Midwifery School, Ahvaz Jundishapur University of Medical Sciences, Ahvaz, Iran.; ^3^Development of Educational Center, Ahvaz Jundishapur University of Medical Sciences, Ahvaz, Iran.; ^4^Diabetes Research Center, Department of Biostatistics and Epidemiology, School of Public Health, Ahvaz Jundishapur University of Medical Sciences, Ahvaz, Iran.

**Keywords:** Cognitive behavioral therapy, Pregnant women, Sexual dysfunction, Sexual self-efficacy, Sex counseling.

## Abstract

**Background:**

Cognitive-behavioral therapy (CBT) is one of the ways to improve an undesirable sexual function.

**Objective:**

The purpose of this study was to investigate the effect of CBT on the sexual function and sexual self-efficacy of pregnant women.

**Materials and Methods:**

In this randomized clinical trial, 36 pregnant women referred to five healthcare centers in Ahvaz, Iran, from December 2016 to January 2017 were enrolled through stratified random sampling in two groups. The case group received counseling based on cognitive behavioral therapy for eight consecutive weeks and the control group received the routine training provided by healthcare staff. Two and four weeks after the end of sessions, both groups completed the Female Sexual Function Index and self-efficacy questionnaires again.

**Results:**

The mean of sexual function and self-efficacy scores in pregnant women in the case and control groups before the intervention did not show a significant difference (p = 0.56). The mean of sexual function and self-efficacy scores of pregnant women in the case and control groups was statistically significant two and four weeks, respectively, after the intervention (p ≤ 0.0001).

**Conclusion:**

The results of this study showed that counseling based on CBT in comparison with the routine training during pregnancy improves the sexual performance and self-efficacy of pregnant women.

## 1. Introduction

Pregnancy is one of the most important courses in women's life (1). The physical, hormonal, and emotional (mental) changes during pregnancy can influence sexual activity as well as sexual relations of couples (2). The studies have shown that there is a connection between pregnancy and sexual dysfunction. The prevalence of sexual dysfunction is very common in pregnant women and can negatively affect a woman's quality of life and couples' relationships (3-4).

The factors influencing sexual behavior during pregnancy can be categorized into biological, psychological, and relational factors (5). Common fears, concerns, and negative attitudes of pregnant women about sex during pregnancy-such as fear of harm to the fetus, pain, premature delivery, abortion, vaginal bleeding, and rapture of membranes-are the most important factors (agents) affecting women's sexual activity during pregnancy (6).

Lack of sexual knowledge is accompanied with enhanced vulnerability and creates a tendency for sexual dysfunction (7).

In nonpregnant women, female sexual dysfunctions have been successfully treated with a spectrum range of interventions including cognitive-behavioral therapy (CBT), sex therapy, psycho education, and couple communication training (4, 8).

Since some of the sexual disorders of women during pregnancy are associated with stress, anxiety, and negative attitudes, it seems that CBT can be considered as one of the effective interventions (9).

Cognitive-behavioral therapists state that cognitive issues are more effective than physiological factors in treating problems. Negative thoughts about sexual activity exacerbate symptoms, so discovering these self-suggestions is helpful in solving sexual problems (10). The CBT is a promising treatment to reduce and improve the symptoms of anxiety and depression experienced during the perinatal period (11). In another study, the results showed that sexual counseling was able to significantly increase the sexual performance of pregnant women from the perspective of themselves and their husbands (12).

On the other hand, sexual self-efficacy is positively correlated with some sexual function subscales. Thus, sexual function in women can be enhanced by increasing women's sexual self-efficacy (13). Sexual self-efficacy refers to the belief of each individual about their ability to be sexually active (13).

In one study, it was discovered that providing cognitive-behavioral counseling to pregnant women during perinatal care can reduce stress and anxiety and increase self-efficacy in choosing the type of delivery.

The CBT with improved sexual self-efficacy and marital satisfaction improves couples' relationships (14).

Because sexual function is part of the health of pregnant women, identifying and eliminating the misconception surrounding sex in pregnant women is essential to improve their sexual health (6). In Iran, despite the existence of effective prenatal care system, sexual counseling during pregnancy has not been fully assessed, and there are gaps in this field (12). Considering that CBT is one of the ways to improve sexual behavior, this study aimed to determine the effectiveness of CBT on the sexual function and sexual self-efficacy of pregnant women in Ahvaz, Iran.

## 2. Materials and Methods

In this randomized clinical trial, pregnant women referred to five healthcare centers in Ahvaz, Iran, from December 2016 to January 2017 were enrolled through stratified random sampling. The five healthcare centers were randomly selected from the centers located in different geographical areas with various levels of socioeconomic status.The sample size was determined according to the results of our pilot study. Given α = 0.05, β = 0.10, 20% attrition, and the total score of the Female Sexual Function Index (FSFI), the sample size was determined to be 18 in each group.

Our inclusion criteria were nulliparous healthy women with low-risk pregnancy, singleton pregnancies, the age range of 18-35 yr, gestational age of 14-16 wk, basic level of literacy, planning a hospital birth, sexual function score of < 26, self-efficacy score < 20, living with the spouse.

Exclusion criteria: having the sexual dysfunction before pregnancy, pregnancy complications (placenta previa, threatened abortion, gestational hypertension, and gestational diabetes), having any vaginal and cervical infections, smokers and alcohol users, use of medications affecting the sexual responses such as antihypertensive, antidepressants, having chronic physical and mental illnesses, having male sexual problems, having recognized physical or mental diseases, having unfortunate events during three months prior to the study.

At first, 60 pregnant women (n = 12/ each center) were randomly selected according to our inclusion and exclusion criteria. After the vaginal examination to rule out organic causes of sexual dysfunction, women were asked to complete the FSFI and sexual self-efficacy questionnaire. Finally, 36 women with self-efficacy score < 20 and sexual function score < 26 were enrolled and randomly assigned into the case and control groups (n = 18/each) using a computer-generated code. In order to maintain confidentiality, the random allocation of subjects were performed by a colleague of the researchers from outside the research team The FSFI is a validated and reliable questionnaire for evaluating the sexual function of women. This questionnaire contains 19 questions that measure women's sexual function in six independent areas of sexual desire, arousal, orgasm, vaginal lubrication, sexual satisfaction, and pain. For scoring, the scores of each field were obtained through the sum of scores of questions in each field and multiplied in the certain factor. The scores of areas of desire, sexual stimulation, vaginal moisture, orgasm, pain, and sexual satisfaction ranged from 0 to 5. Zero indicated a person having no sexual activity for the past four weeks. The total score of FSFI is ranged from 2 to 36. Scores > 26 was considered as a good sexual function (15). The validity and reliability of FSFI was assessed and approved by Fakhri *et al*. (16).

Another instrument used in this study was Schwartzer's sexual self-efficacy questionnaire, whose validity and reliability was constructed by Vaziri and Lotfi Kashani (17). The questionnaire included 10 questions, and each question could be scored between 0 (for not true) and 30 (that is true). The results were then divided into the following three categories: low self-efficacy (score < 10), moderate (scores 10-20), and high (score > 20). The reliability of the self-efficacy questionnaire has been reported using Cronbach's alpha (86%), and the validity of the questionnaire of sexual self-efficacy has been performed using content validity method.

Study participants received eight 45-minute counseling per week (n = 6 in each in sessions). Counseling sessions for both case and control groups was planned on different days.

The CBT sessions are prepared from evidence-based research (18-24). In order to validate the contents of the counseling sessions and the treatment method, the opinions of 10 experts (psychologists, gynecologist, and midwife) familiar with CBT were used and their comments were considered and applied.

The contents of the consultation and treatment sessions are listed in table I. A clinical psychologist supervised all training sessions and the researcher who (the first author) trained pregnant women worked under close supervision of psychologist. The time scheduled for group counseling was based on the age of the pregnancy. In the control group, routine training during prenatal care was performed. Two and four weeks after the last session, the standard sexual function and self-efficacy questionnaires were, respectively, completed by all the participants. After following-up on the study, specialized counseling for mothers in the control group was performed.

**Table 1:** Content of the counseling sessions


**Sessions **	**Content**
**Session 1 **	• *Interview*. Identify the underlying factors (factors that make a person vulnerable to sexual issues). Detector factors (factors affecting sexual problems). Continuity or maintenance agents (psychological responses to sexual issues, feedback, and other stresses that cause problem stiffness or deterioration) • Assignment: Practice identifying cognitive distortions using intellectual card
**Session 2**	• *Formulation*. At this stage, the therapist should strive to balance the contribution of each side to the problem, in order to emphasize the need for the parties to participate in the treatment process. The therapist should also highlight the positive aspects of the couple's relationship • Check assignments: Renewal of cognitive falsification
**Session 3**	• *Cognitive reconstruction*. The most important action of cognitive reconstruction techniques is to train the patterns of thinking more adapted to individuals. To help them discover the negative and distorted patterns of thought, and thereby identify the damaging effects of these patterns of thought, and replace the more consistent and correct patterns of thinking rather than their ineffective cognitive responses • Assignment: Practice cognitive restructuring
**Session 4**	• *Sexual knowledge*. Provide sexual information and the benefits of sexual relationships in life-long building and improving relationships and early sexual intercourse and having a baby with more relaxation • Check assignment: Cognitive restructuring
**Session 5**	• *Non-sexual sensation*. In this way, couples spend only a few weeks, noting their proximity and reaching the orgasm, only to stroke and massage inappropriate places • Assignment: Practice avoiding inappropriate thoughts
**Session 6**	• *Sexual* *sensation*. At this stage, they can enjoy the sexual areas and, in this regard, they stroke and massage each other's sexual areas, but at this stage the goal is sexual pleasure and arousal, not getting orgasm • Check assignment: Coping, and avoiding inappropriate behaviors
**Session 7**	• *Teaching how to have sex during pregnancy:* The best condition for a pregnant woman is a situation where a pregnant woman is up or sideways. The other favorable positions in pregnancy are: Sleeping sideways, where the penis should be penetrated from the back to the vagina, both sitting face to face and female foot on the foot of the spouse, woman bending over and the penis being penetrated from the back to the vagina • Assignment: Practice avoiding inappropriate behaviors
**Session 8**	• *Evaluate the result*. Assessing pregnant women to determine which therapeutic goals are achieved. • Check assignment: Prevention of approaches to fight back

### Ethical consideration

After explanation of the study objective and intervention methods, a written informed consent form was obtained from each participant. This study proposal was approved by the Ethics Committee of Ahvaz Jundishapur University of Medical Sciences, Ahvaz, Iran (Code: IR.AJUMS.REC.1395.484).

### Statistical analysis

Data were analyzed using the SPSS software version 20.0 (Statistical Package for the Social Sciences, version 20.0, SPSS Inc, Chicago, Illinois, USA). The significance level was considered as p < 0.05. Descriptive statistics were used to provide the indicators (mean and standard deviation) and analytical statistics including Student's *t* test and paired *t* test were used for normal variables. The Kolmogorov-Smirnov test was used to verify that the data from the test run have a normal distribution. The Mann-Whitney test was used in case of variables that were not normally distributed.

## 3. Results

**Figure 1:**
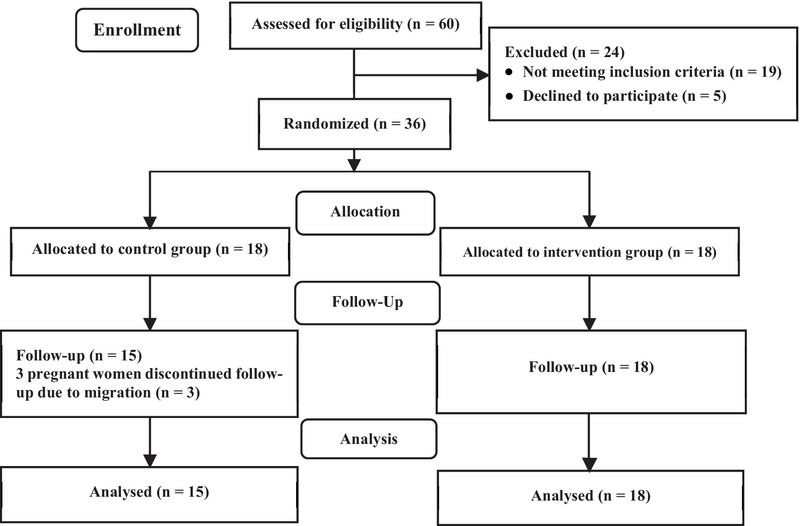
Flowchart of the progress through the phases of the trial.

From 60 pregnant women enrolled initially in this study, 24 were excluded for the reasons mentioned in Figure 1 and thirty six women were assigned to the case and control groups (n = 18/each). During the follow-up period, three women from the control group withdrew due to migration. Finally, the data of 18 participants in the case group and 15 women in the control group were analyzed (Figure I).

No significant differences were observed in two groups in the terms of age, educational level, and gestational age. The demographic characteristics of the participants in two groups were presented in table II.

Self-efficacy scores before intervention in the case group was 8.77 ± 2.98 and in the controls was 9.40 ± 3.75 (p = 0.56). There was a significant difference between the case and control groups, two weeks after intervention (24.5 ± 3.16 versuse 10.7 ± 3.67, respectively, p = 0.0001) and also, four weeks after intervention (24.22 ± 2.66 versus 10.4 ± 2.5, p = 0.0001).

There was no significant difference in the terms of sexual function in both groups before the intervention (p = 0.71). Also, there were no significant differences between the groups in all domains of sexual function (Table III).

**Figure 2:**
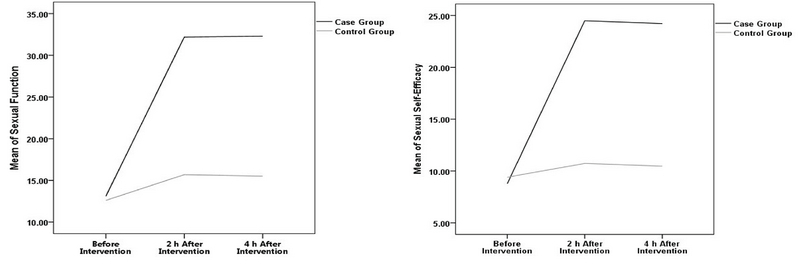
A: Sexual function of the case and control groups before the intervention and two and four weeks after intervention, B: Sexual self-efficacy of the case and control groups before intervention and two and four weeks after intervention.

The results indicated that after the counseling sessions, the mean sexual function index and sexual self-efficacy of the intervention group significantly increased as compared to the control group (p = 0.001; Figures 2A, 2B).

Based on the findings, two wk. after intervention, the mean sexual function index in the case group significantly increased compared to the controls (p = 0.001). Four weeks after intervention, a significant difference was observed between the two groups in the terms of sexual function (p = 0.001). Furthermore, the sexual function index did not significantly change two and four weeks after the cognitive-behavioral training (p = 0.84; Table III; Figure 2A).

Two weeks after intervention, the sexual self-efficacy showed a significant difference between two groups (p = 0.001). In addition, four weeks after cognitive-behavioral counseling, there was a significant increase in mean scores of sexual self-efficacy in the case group (Figure 2B).

After the counseling sessions, the control group obtained very low levels of function improvement in all domains and total sexual function. In the case group, however, the total sexual function score and scores of all other domains increased. It indicates the effectiveness of consultation in identifying problems among women, which was leading to a significant difference between the two groups (p < 0.001; Table IV).

**Table 2:** Demographic characteristics of the participants in two study groups


**Variables** **Groups $1^{1}$Groups**		**Case (n = 18)**	**Control (n = 15)**	**P-value**
Age (yr)*		5.0 ± 21.8	2.9 ± 19.3	0.19
Husbands' age (yr) *		4.3 ± 27.3	0.7 ± 22.5	0.16
Gestational age (wk) *		8.7± 14.9	9.0 ± 15.1	0.54
Women's education**				
	**Illiterate**	0 (0)	1 (5.5)	0.39
	**Diploma and high school**	12 (66.6)	12 (66.6)	
	**Bachelor's degree and higher**	6 (33.3)	5 (27.7)	
Men's education**				
	**Illiterate**	2 (11.1)	0 (0)	0.30
	**Diploma and high school**	12 (66.6)	12 (66.6)	
	**Bachelor's degree and higher**	4 (22.2)	6 (33.3)	
Economic status**				
	**< 1 million**	9 (50)	8 (44.4)	0.65
	**Between 1 and 2 million**	8 (44.4)	8 (44.4)	
	**> 2 million**	1 (5.5)	2 (11.1)	
*Data presented as Mean ± SD (independent-samples *t* test), **Data presented as n (%) (Mann-Whitney test)				

**Table 3:** Comparison of female sexual function index (FSFI) scores before and after intervention between case (n = 18) and control (n = 15) groups


**Functional domains**	<Before intervention*		<Two weeks after intervention*		<Four weeks after intervention*		
	Case	**Control**	**P-value**	Case	**Control**	**P-value**	Case	**Control**	**P-value**
**Sexual desire**	2.91 ± 0.85	2.64 ± 1.25		0.25	5.04 ± 0.64		3.04 ± 1.0	0.0001		4.06 ± 8.64	3.0 ± 3.87	0.0001
**Arousal**	2.02 ± 1.76	1.98 ± 1.29	0.82	5.45 ± 0.31	2.48 ± 1.23	0.0001	5.46 ± 0.34	2.54 ± 1.20	0.0001
**Orgasm**	1.65 ± 1.33	1.73 ± 1.07	0.94	5.21 ± 0.45	2.45 ± 1.10	0.0001	5.18 ± 1.46	2.30 ± 1.03	0.0001
**Satisfaction**	2.22 ± 1.15	1.94 ± 0.75	0.41	5.62 ± 0.36	2.69 ± 0.91	0.0001	5.64 ± 0.38	2.78 ± 0.89	0.0001
**Pain**	2.13 ± 1.69	1.84 ± 1.12	0.39	5.6 ± 0.58	2.48 ± 1.16	0.0001	5.45 ± 0.57	2.60 ± 1.20	0.0001
**Vaginal lubrication**	2.03 ± 1.51	2.04 ± 4.37	0.87	5.38 ± 0.54	2.36 ± 1.09	0.0001	5.43 ± 0.58	2.37 ± 1.09	0.0001
**Total**	12.57 ± 6.72	12.20 ± 4.54	0.71	32.18 ± 1.71	15.68 ± 5.46	0.0001	32.28 ± 1.89	15.51 ± 1.89	0.0001
Values are expressed as Mean ± SD, Mann-Whitney test									

**Table 4:** Comparison of the level of sexual self-efficacy and sexual function of stage 1, 2, and 3 in the case (n = 18) and control (n = 15) groups


[2.1in,lr]**Variables** **Significance level of steps**	**Step 1 and 2**		**Step 1 and 3**		**Step 2 and 3**	
	Case	**Control**	Case	**Control**	Case	**Control**
**Sexual self-efficacy**	0.0001	0.16	0.0001	0.036	0.79	0.9
**Sexual function**	0.0001	0.118	0.0001	0.42	0.84	0.74
Step 1: Before the intervention; Step 2: Two weeks after the intervention; Step 3: Four weeks after intervention, Wilcoxon test						

## 4. Discussion

The results of this study showed that the counseling based on CBT in comparison with the routine training during pregnancy improves sexual function and self-efficacy of pregnant women.

Sexual dysfunction during pregnancy is characterized by disturbances in sexual desire and in the psycho-physiological changes. It seems obvious that psychological factors play an important role in sexual dysfunction in pregnant women. CBT mainly through the change of maladaptive cognitions leads to change in emotional distress and problematic behaviors. Research suggests that CBT is effective in treating sexual dysfunction, especially in women, through the change of these negative schemas (25).

The biological changes that occur during pregnancy may have a direct impact on sexual function, and in addition to an effect of the hormone, emotional changes can also include change in lifestyle and image of you, as well as changes in sexuality and sexual behavior (26).

Lack of knowledge about changes in pregnancy can lead to sexual responses. Babazadeh and colleagues reported that quite often pregnant women do not receive any information on sexual issue related to pregnancy during prenatal care (27).

Afshar and Colleagues stated an improvement in sexual function of pregnant women after use of an educational program. In this study, a relationship was observed between sexual knowledge and sexual function. Most couples did not have adequate information about sexual knowledge during pregnancy. In addition, the importance of sex education with a positive impact on the sexual life of pregnant women was shown (28). Contrary to this result, it has been reported that the sexual behavior of pregnant women who have undergone sex education did not show a statistically significant difference with those of the control group (29). The reason for the different in this results could be the specifications of the study population, tools used in the study, content and number of education sessions.

Concerns, fears, stress, anxiety and negative attitudes during pregnancy reduce satisfaction and performance of sexual in pregnant women (7). Lowndes and colleagues reported that CBT through brief guided self-help can reduce the symptoms of anxiety and depression in pregnant women during the third trimester of pregnancy (30). The study of Sabetnejad and co-worker with the aim of “the effectiveness of CBT and fluoxetine on sexual function of women with obsessive-compulsive disorder" showed that sexual performance after OCD treatment in the CBT group was significantly improved compared to the fluoxetine group. Researchers reported that CBT can be used for the improvement of sexual function in clinical practices (31).

Vakilian and colleagues pointed out that cognitive-behavioral counseling during pregnancy plays an important role in decreasing the misconceptions about sexual intercourse during pregnancy in pregnant women (7). Riazi and colleagues demonstrated that sex education has a positive and significant effect on correcting dysfunctional misconceptions about sex in pregnancy and thus improve the quality of sex in pregnant women (32).

A study by Navidian and colleagues in Iran, aimed at “Investigating the effect of group sex counseling on traditional perceptions and attitudes of pregnant women," found that group sex counseling had a positive effect on improving pregnant women's traditional perceptions and attitude toward sex during pregnancy (33).

Ter Kuile co-worker reported that CBT can improve sexual dysfunctions in women (34).

In this regard, McCabe evaluates a cognitive behavior therapy program for people with sexual dysfunction. He found that the treatment led to “lower levels of sexual dysfunction, more positive attitudes toward sex, perceptions that sex was more enjoyable, fewer affected aspects of sexual dysfunction in their relationship, and a lower likelihood of perceiving themselves as a sexual failure" (35). These results were in line with our finding.

Also, Saboula and colleagues reported that CBT as an important nursing intervention is an effective therapy in managing dyspareunia for decreasing sexual pain severity, improving sexual performance, marital adjustment, psychosocial state (36). Findings from the study of Robin co-worker suggest that psychosocial treatments for vulvodynia are effective. CBT, a directed treatment approach that involves learning and practice of specific pain-relevant coping and self-management skills yielded better outcomes and greater patient satisfaction than a less directive approach (37). Ramesh and co-worker conducted a study aimed at “Effectiveness of combination of CBT and biofeedback on vaginismus patients' sexual function and marital status." They reported that the use of CBT and biofeedback combinational therapy is effective in improving vaginismus, sexual function, and marital status (38).

Navidian and colleagues investigated the impact of sex counseling on sexual response of pregnant women in Zahedan. The results showed that in the intervention group, counseling was able to improve sexual function in six dimensions including sexual desire, intercourse frequency, satisfaction, arousal, orgasm, and sexual quality (12).

The findings of our study is in accordance with the results of the mentioned studies. It seems that counseling based on cognitive-behavioral approach can significantly improve the female sexual function during pregnancy.

In contrast to these results, the study of Vakilian and colleagues in Iran, aimed at evaluating the effect of cognitive-behavioral approach to sexual counseling on the female sexual performance during pregnancy showed that “counseling failed to overcome the biological and psychological changes during pregnancy and could not improve the sexual function of the participants"(39).

The results of the mentioned study are not consistent with our findings. The differences in the number of participants and the cognitive-behavioral counseling approach may be the reason for the differences in the results of two studies.

The results of all studies in this area were based on a comparison of the mean score of sexual performance; whereas in the study of the Vakilian and colleagues, the final result of the study was based on the percentage of participants who achieved a sexual performance mean score > 26 followed by counseling (39). In the mentioned study, Although the mean score of sexual function in pregnant women was higher in the intervention group compared to the control group, they reported that only 36.5% of the participants achieved a score > 26 and the rest obtained a score < 26, which was within the inappropriate range.

In this regard, it seems that improving the mean score of sexual function in 36.5% of pregnant women is a valuable outcome. Furthermore, the mean score of sexual function among the participants of this study increased from 17.40 ± 9.83 to 26.81 ± 3.57, which is satisfactory in pregnant women given the hormonal and biological changes specific to pregnancy. Therefore, it seems reasonable that counseling in pregnancy is necessary to improve sexual function in pregnant women.

As stated earlier, the results of this study showed that counseling based on CBT in comparison with the routine training during pregnancy improves sexual self-efficacy of pregnant women.

A study by Mohamadi zadeh and Moradi joo to examine the effect of cognitive counseling on women's self-efficacy showed that this kind of counseling approach improves their self-efficacy, which is consistent with this study (40). Sexual self-efficacy includes every person's belief in their ability to perform sexually effectively. In fact, sexual self-efficacy predicts sexual function (41). The results of the study by Jamali and colleagues showed that cognitive-behavioral intervention improves couples' relationships by improving sexual self-efficacy and marital satisfaction (14). In another study conducted in the healthcare centers of Shahid Beheshti University of Medical Sciences, Ramezani and colleagues showed a significant relationship between self-esteem and sexual dysfunction in women, especially in those with low self-esteem that presented greater sexual dysfunction (42). These results were in line with our finding.

### Limitation

One of the limitations of the present study was the absence of spouses in counseling sessions. Given cultural sensitivities and issues around sexuality in our study, pregnant women participated in counseling sessions without their spouses. However, they were asked to educate their husbands about the topics they learned during the counseling sessions.

Due to cultural reasons and the sensitivity of the topic, it is possible that the participants would have avoided giving correct information. The lack of communication between the researcher and the husbands of pregnant women was one of the other limitations of this study.

One of the strengths of our study is conducting cognitive behavioral counseling with emphasis on improving sexual self-efficacy in pregnant women.

## 5. Conclusion

In this research, the data pointed to the positive impact of CBT on sexual function and sexual self-efficacy during pregnancy.

Therefore, it is recommended that health managers and planners through midwifery counseling promote sexual health of pregnant women during prenatal care.

##  Recommendation

It is recommended to determine the effect of couples' cognitive behavioral counseling on marital satisfaction during pregnancy and determine the effect of CBT on sexual function and sexual self-efficacy in men.

##  Conflict of Interest 

None to declare.
